# Age-dependent transition from islet insulin hypersecretion to hyposecretion in mice with the long QT-syndrome loss-of-function mutation Kcnq1-A340V

**DOI:** 10.1038/s41598-021-90452-8

**Published:** 2021-06-10

**Authors:** Anniek F. Lubberding, Jinyi Zhang, Morten Lundh, Thomas Svava Nielsen, Mathilde S. Søndergaard, Maria Villadsen, Emil Z. Skovhøj, Geke A. Boer, Jakob B. Hansen, Morten B. Thomsen, Jonas T. Treebak, Jens J. Holst, Jørgen K. Kanters, Thomas Mandrup-Poulsen, Thomas Jespersen, Brice Emanuelli, Signe S. Torekov

**Affiliations:** 1 Department of Biomedical Sciences, Faculty of Health and Medical Sciences, University of Copenhagen, Blegdamsvej 3B, 2200 Copenhagen, Denmark; 2 Novo Nordisk Foundation Center for Basic Metabolic Research, Faculty of Health and Medical Sciences, University of Copenhagen, Copenhagen, Denmark

**Keywords:** Physiology, Endocrinology, Diabetes, Cardiovascular diseases, Arrhythmias

## Abstract

Loss-of-function (LoF) mutations in *KCNQ1*, encoding the voltage-gated K^+^ channel K_v_7.1, lead to long QT syndrome 1 (LQT1). LQT1 patients also present with post-prandial hyperinsulinemia and hypoglycaemia. In contrast, *KCNQ1* polymorphisms are associated with diabetes, and LQTS patients have a higher prevalence of diabetes. We developed a mouse model with a LoF *Kcnq1* mutation using CRISPR-Cas9 and hypothesized that this mouse model would display QT prolongation, increased glucose-stimulated insulin secretion and allow for interrogation of K_v_7.1 function in islets. Mice were characterized by electrocardiography and oral glucose tolerance tests. Ex vivo, islet glucose-induced insulin release was measured, and beta-cell area quantified by immunohistochemistry. Homozygous mice had QT prolongation. Ex vivo, glucose-stimulated insulin release was increased in islets from homozygous mice at 12–14 weeks, while beta-cell area was reduced. Non-fasting blood glucose levels were decreased at this age. In follow-up studies 8–10 weeks later, beta-cell area was similar in all groups, while glucose-stimulated insulin secretion was now reduced in islets from hetero- and homozygous mice. Non-fasting blood glucose levels had normalized. These data suggest that K_v_7.1 dysfunction is involved in a transition from hyper- to hyposecretion of insulin, potentially explaining the association with both hypoglycemia and hyperglycemia in LQT1 patients.

## Introduction

Voltage-gated K^+^ (K_v_) channels play an important physiological role in repolarization of the action potential in a variety of tissues^1,2^. *KCNQ1* encodes the K_v_7.1 K^+^ channel pore-forming subunit, which co-assembles with *KCNE1* encoded auxiliary subunits to form the channel responsible for the slow delayed rectifier K^+^ current (*I*_Ks_) in cardiomyocytes^2,3^. Reduced *I*_Ks_ results in long-QT syndrome (LQTS), a cardiac disorder characterized by delayed repolarization, a prolonged QT interval in the electrocardiogram (ECG), and increased risk of ventricular arrhythmia and sudden cardiac death^4^. Loss-of-function (LoF) mutations in *KCNQ1* represent the most common cause of LQTS, occurring in 40–50% of all LQTS patients, and is termed LQT1^5^. *KCNQ1* is also expressed in a variety of endocrine tissues such as human pancreatic beta-cells^6^, alpha-cells^7^, the gastrointestinal tract^8^ and hypothalamic GNRH secreting neurons^9^. While a gain-of-function mutation in *KCNQ1* was associated with reduced insulin response to oral glucose^10^ LQTS patients with LoF mutations in *KCNQ1* present with hyperinsulinemia and subsequent hypoglycemia after an oral glucose challenge^11^. Similar findings were made in patients with LoF mutations in *KCNH2*, encoding K_v_11.1 (hERG), the second most common cause of LQTS^12^. This may be hazardous, since glucose ingestion, as well as hypoglycaemia, prolongs the QT interval in healthy individuals^12–14^ and LQTS patients^12,14^, which could potentially lead to cardiac arrhythmias^15^. In paediatric patients, this presents a significant clinical problem since the common use of beta-adrenergic blockers may aggravate hyperinsulinemia^16^ by blocking epinephrine-mediated inhibition of insulin secretion.

In line with the human phenotype, pharmacological inhibition of K_v_7.1 increased insulin secretion upon oral glucose in mice^17^, while overexpression of wild-type K_v_7.1 in the MIN6 mouse beta-cell line resulted in reduced glucose-stimulated insulin secretion^18^. In pancreatic beta-cells, high extracellular glucose initiates insulin secretion by closure of ATP-dependent K^+^ channels, which depolarize the membrane potential, leading to action potential firing, opening of voltage-gated Ca^2+^ channels, and increased intracellular Ca^2+^ concentration^19^. This subsequently leads to Ca^2+^-dependent exocytosis of insulin. Several K_v_ channels are expressed in the pancreatic beta-cell and contribute to repolarization and hyperpolarization of the membrane potential, halting further insulin secretion^19,20^. It remains unclear precisely what role K_v_7.1 plays in insulin secretion.

Interestingly, genome-wide association studies (GWAS) have revealed that common variants in *KCNQ1* are associated with the development of type 2 diabetes (T2D)^6,21^. A study in human pancreatic beta-cells showed that some of these susceptibility variants near *KNCQ1* were accompanied by either reduced depolarization-induced insulin secretion or impaired insulin granule docking^22^. It is unknown whether these variants influence K_v_7.1 channel function; however, a recent Danish retrospective cohort study showed that LQTS patients have a higher prevalence of diabetes, suggesting reduced K^+^ currents could be involved^23^. Moreover, in mice, a mutation in the noncoding region of the *Kcnq1* locus on the paternal allele was shown to reduce pancreatic beta-cell mass through epigenetic modification of cell cycle inhibitor cyclin-dependent kinase inhibitor 1C (*Cdkn1c*)^24^. Genetic polymorphisms of *KCNQ1*, as well as methylation at the *KCNQ1* locus, have also been associated with altered insulin sensitivity^25,26^. *Kcnq1* homozygous knockout (KO) mice showed increased insulin sensitivity revealed by lower blood glucose and insulin levels during fasting^27^. These observations emphasize that studies to provide a unifying model of the precise role of K_v_7.1 in pancreatic beta-cell function and glucose homeostasis are in demand.

To study the role of K_v_7.1 in insulin secretion and glucose homeostasis in greater detail, we developed a novel LQTS mouse model using clustered regularly interspaced short palindromic repeats (CRISPR)-Cas9 gene editing technique^28^ to insert the LQT1-causing LoF mutation *Kcnq1*-A340V. A340 is the residue in mice corresponding to A341 in humans. The disease-causing A341V mutation, located on transmembrane helix S6^29^, leads to a clinically severe QT phenotype^30^, and in heterozygous conditions leads to a 50% reduction in *I*_Ks_^31–34^. We hypothesized that our mouse model would recapitulate the human phenotype, such as prolonged QT interval, along with increased glucose-stimulated insulin secretion in vivo, and allow for interrogation of the intrinsic function of K_v_7.1 in islets.

## Results

### Functional confirmation of *Kcnq1* loss-of-function mutation

**Figure 1:**
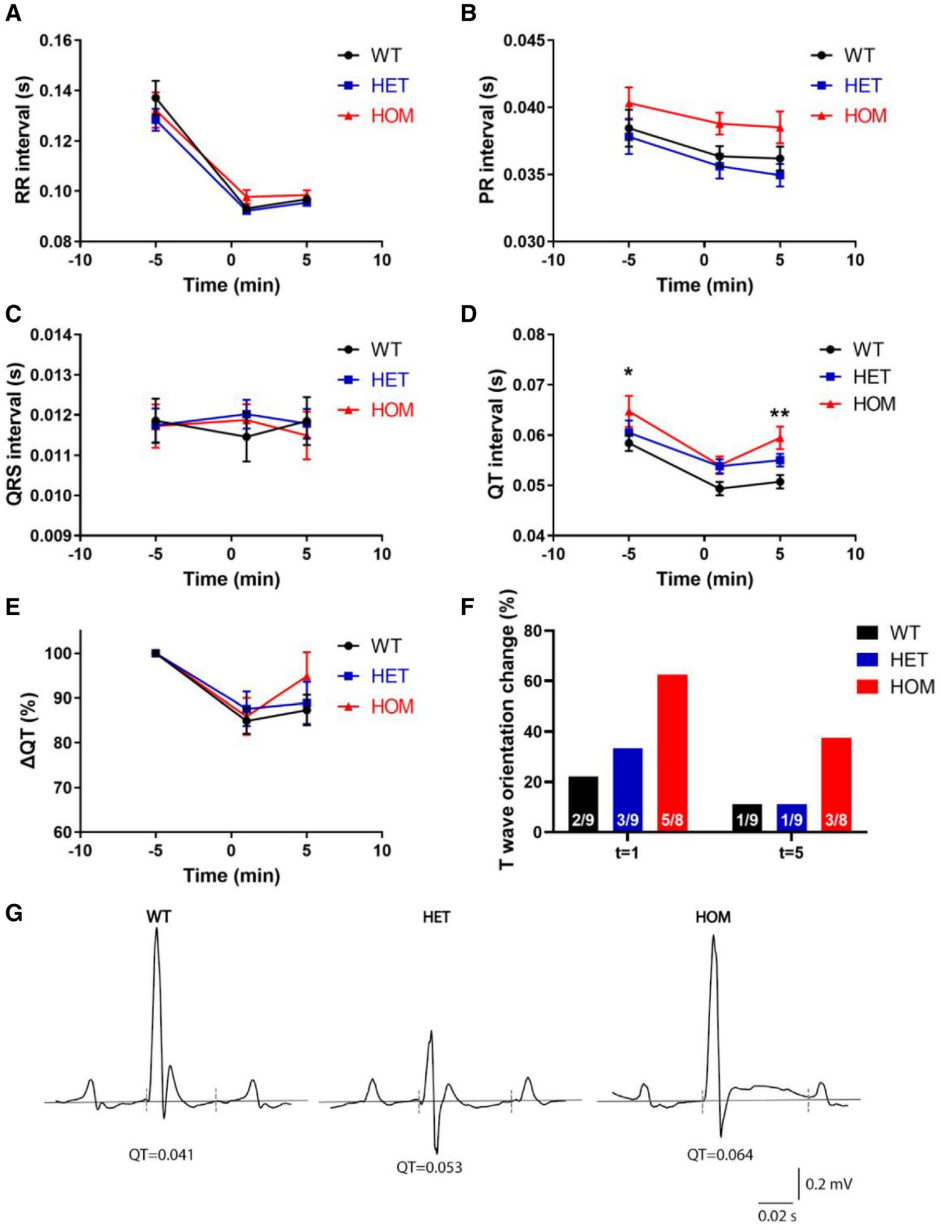
Electrocardiograms during anaesthesia confirm longer QT interval. (**A**–**D)** RR interval (**A**), PR interval (**B**), QRS duration (**C**) and QT interval (**D**) at baseline and after isoprenaline administration in WT (n = 9), HET (n = 9) and HOM (n = 8) mice. (**E**) QT (%) change from baseline. (**F**) Isoprenaline induced changes in T wave orientation in a subset of WT and HET mice and the majority of HOM mice 1 min after administration, which subsided 5 min after administration in the majority of cases. (**G)** Representative traces five minutes after isoprenaline injection. The dashed lines indicate where QT was measured using the tangent approach. Note that in this HOM mouse, the T wave was not only prolonged, but also became positive, illustrating the T wave orientation changes of **F**. (**A**–**E)** tested with two-way ANOVA with Dunnett’s multiple comparisons testing. *: WT versus HOM, *P* < 0.05, **: WT versus HOM, *P* < 0.01. ANOVA, analysis of variance; HET, heterozygous; HOM, homozygous; WT, wild-type.

The ECG was recorded under anaesthesia to functionally confirm reduced K_v_7.1 function. RR intervals were comparable between genotypes at baseline and after isoprenaline administration (Fig. 1A), as were PR interval (Fig. 1B; *P* = 0.058) and QRS duration (Fig. 1C; *P* = 0.96) At baseline, the QT interval was prolonged in homozygous (HOM) mice compared to wild-type (WT; *P* = 0.03) with a QT interval of 58 ± 2, 60 ± 2 and 65 ± 4 ms in WT, heterozygous (HET) and HOM, respectively (Fig. 1D). The differentiation of QT length became more distinct by injection of isoprenaline, giving a QT interval of 51 ± 1, 55 ± 1 and 60 ± 2 in WT, HET and HOM, respectively (WT vs HOM: *P* = 0.005; Fig. 1D,E). In the majority of HOM mice, the QT interval was not only increased, but the T wave also became positive after isoprenaline administration (Fig. 1F), as illustrated by representative ECG traces (Fig. 1G). No arrhythmias were observed during the isoprenaline challenge. These data support that the *KNCQ1* mutation led to a reduction in K_v_7.1 function in these mice.

### *Kcnq1* loss-of-function mutation does not affect in vivo glucose homeostasis

**Figure 2:**
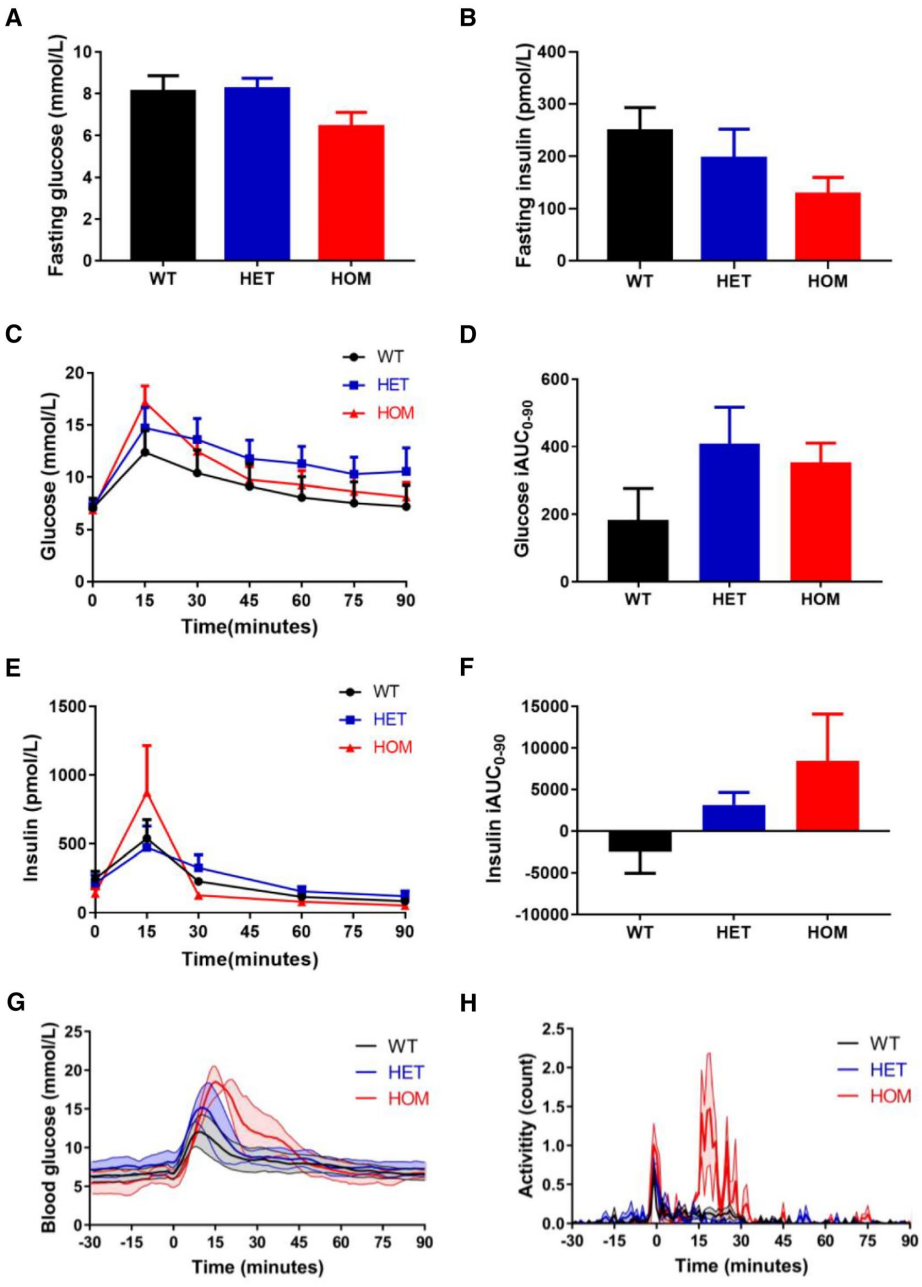
Blood glucose and plasma insulin levels during 90 min oral glucose tolerance test. (**A**) The fasting whole blood glucose levels were measured from tail blood samples after five hours of fasting in WT (n = 10), HET (n = 17) and HOM (n = 15) mice. (**B**) The fasting insulin concentrations were measured from retro-orbital blood samples in WT (n = 6), HET (n = 9) and HOM (n = 8) mice. (**C**) Whole blood glucose levels were measured from tail blood samples during OGTT in WT (n = 6), HET (n = 9) and HOM (n = 8). (**D**) Incremental AUC values (min*mmol/L) of whole blood glucose were calculated. (**E**) Plasma insulin concentrations were measured from retro-orbital blood samples during OGTT in the same mice. (**F**) Incremental AUC values (min*pmol/L) of plasma insulin levels were calculated. (**G**) Blood glucose levels were continuously recorded by telemetry during a 90 min OGTT in WT (n = 5), HET (n = 3) and HOM (n = 2) mice. (**H**) Activity levels, simultaneously recorded during OGTT, revealg high activity in HOM mice. (**A, B, D**, **F**) tested with one-way ANOVA with Dunnett’s multiple comparisons testing. ANOVA, analysis of variance; AUC, area under the curve; HET, heterozygous; HOM, homozygous; OGTT, oral glucose tolerance test; WT, wild-type.

To analyse the metabolic response to oral glucose, oral glucose tolerance tests (OGTTs) were performed in the mice at 9–11 weeks of age. Fasting blood glucose was comparable in the three groups (Fig. 2A; *P* = 0.08), as was fasting plasma insulin (Fig. 2B; *P* = 0.25). The resulting HOMA-IR index of 12 ± 2.3, 13 ± 3.7 and 6 ± 1.6 in WT, HET and HOM mice was also comparable (*P* = 0.17). During OGTT (Fig. 2 C,D), the insulin response was comparable in WT, HET and HOM mice (iAUC_0-90_: *P* = 0.18; Fig. 2E,F). However, handling associated with the OGTT caused high levels of potentially stress-induced physical activity in the HOM mice, as exemplified in a subset of mice that underwent OGTT during continuous telemetric blood glucose monitoring (Fig. 2G,H), which could pose as a confounder of in vivo glucose homeostasis.

### Feeding prolongs QT interval and increases premature ventricular contractions in *Kcnq1* A340V mice

**Figure 3:**
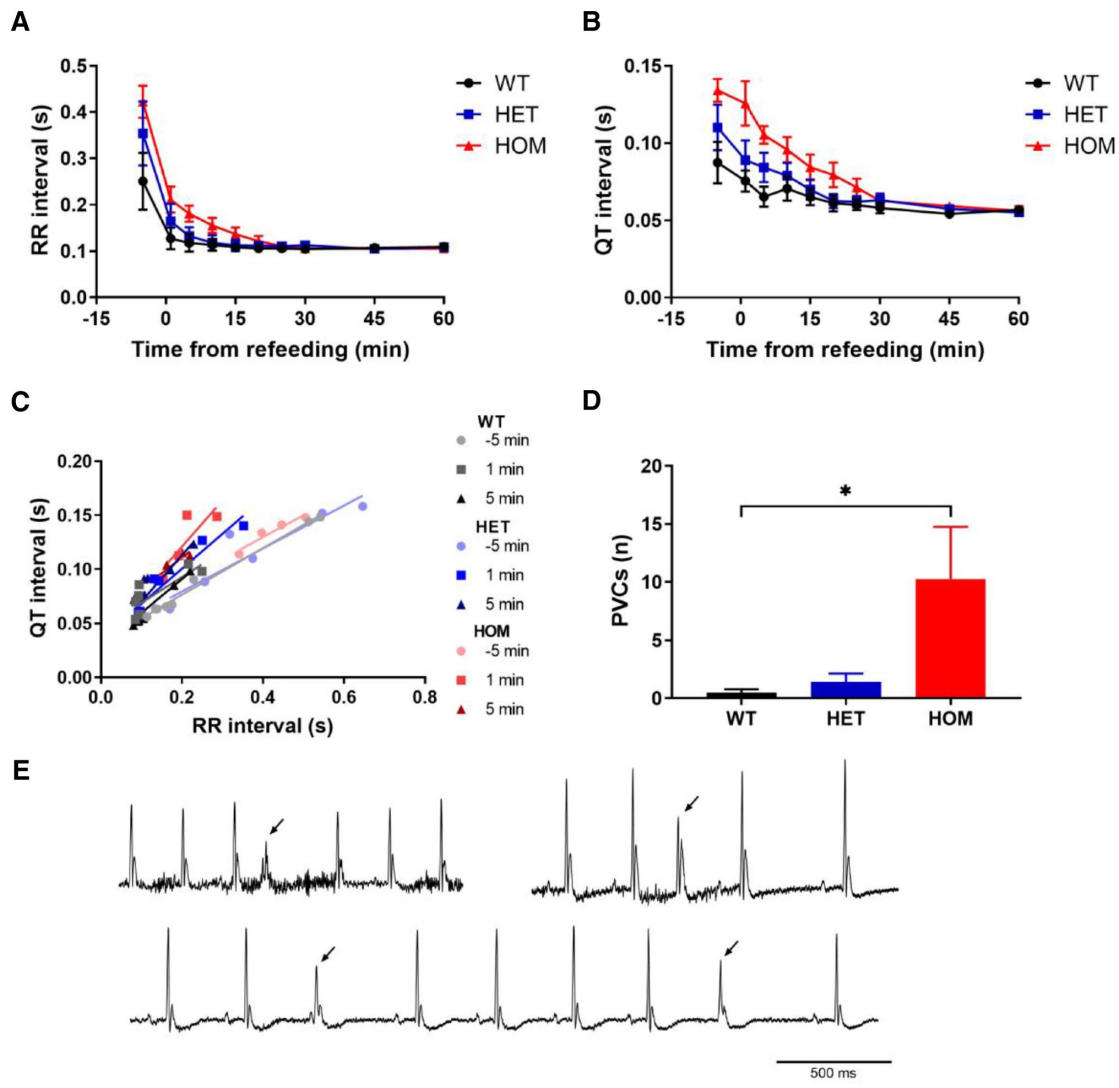
Electrocardiogram telemetry during refeeding. WT (n = 8), HET (n = 7) and HOM (n = 4) mice were fasted overnight for 18 h, after which they were re-fed. (**A**, **B**) RR interval **(A)** and QT interval **(B)** dropped immediately upon refeeding. (**C**) RR/QT relationships were plotted and revealed a change after refeeding in HET and HOM mice, but not in WT mice. (**D**) During the first 30 min after refeeding, PVCs were counted, revealing an increased number of PVCs in HOM mice. (**E**) Representative ECG traces with PVCs in three different mice (PVCs indicated by arrowheads). Analyzed with Kruskal–Wallis test with Dunn's multiple comparisons testing. *: WT versus HOM, *P* < 0.05. HET, heterozygous; HOM, homozygous; PVC, premature ventricular contraction; WT, wild-type.

In human LQTS patients, oral glucose after overnight fasting exacerbates their long QT, which could potentially increase the risk of arrhythmia^14^. To examine this link, ECG telemetry devices were implanted in the mice, allowing for continuous ECG measurements in the awake, undisturbed animal. The ECG was subsequently monitored during overnight fasting and reintroduction of food. All mice began to eat immediately. Upon refeeding, the RR interval dropped, and the QT interval shortened (Fig. 3A,B), as did PR interval and QRS duration (Figure S3). The QT/RR interval relationships (Fig. 3C) were comparable in all groups during fasting (comparison of slopes: *P* = 0.87; comparison of y-intercepts: *P* = 0.19). In WT mice the QT/RR relationship did not change from fasting to refeeding, while in HET and HOM mice the QT/RR relationship shifted upwards (Fig. 3C), indicating an increased QT interval at a given RR interval. This resulted in QT/RR relationships with comparable slopes between groups (*P* = 0.43), but with significantly different y-intercepts (*P* = 0.04). To assess if this metabolic challenge increased the propensity for arrhythmias, premature ventricular contractions (PVCs) were manually counted during the first 30 min after refeeding (Fig. 3D). While PVCs were rare in the WT and HET mice, the number of PVCs was significantly increased in the HOM mice (*P* = 0.01 compared to WT). Examples of PVCs are depicted in Fig. 3E. Taken together, these data indicate that metabolic challenges may evoke ventricular repolarization disturbances in mice lacking functional K_v_7.1.

### Increased ex vivo insulin secretion in islets from young mice with *Kcnq1* A340V mutation

**Figure 4:**
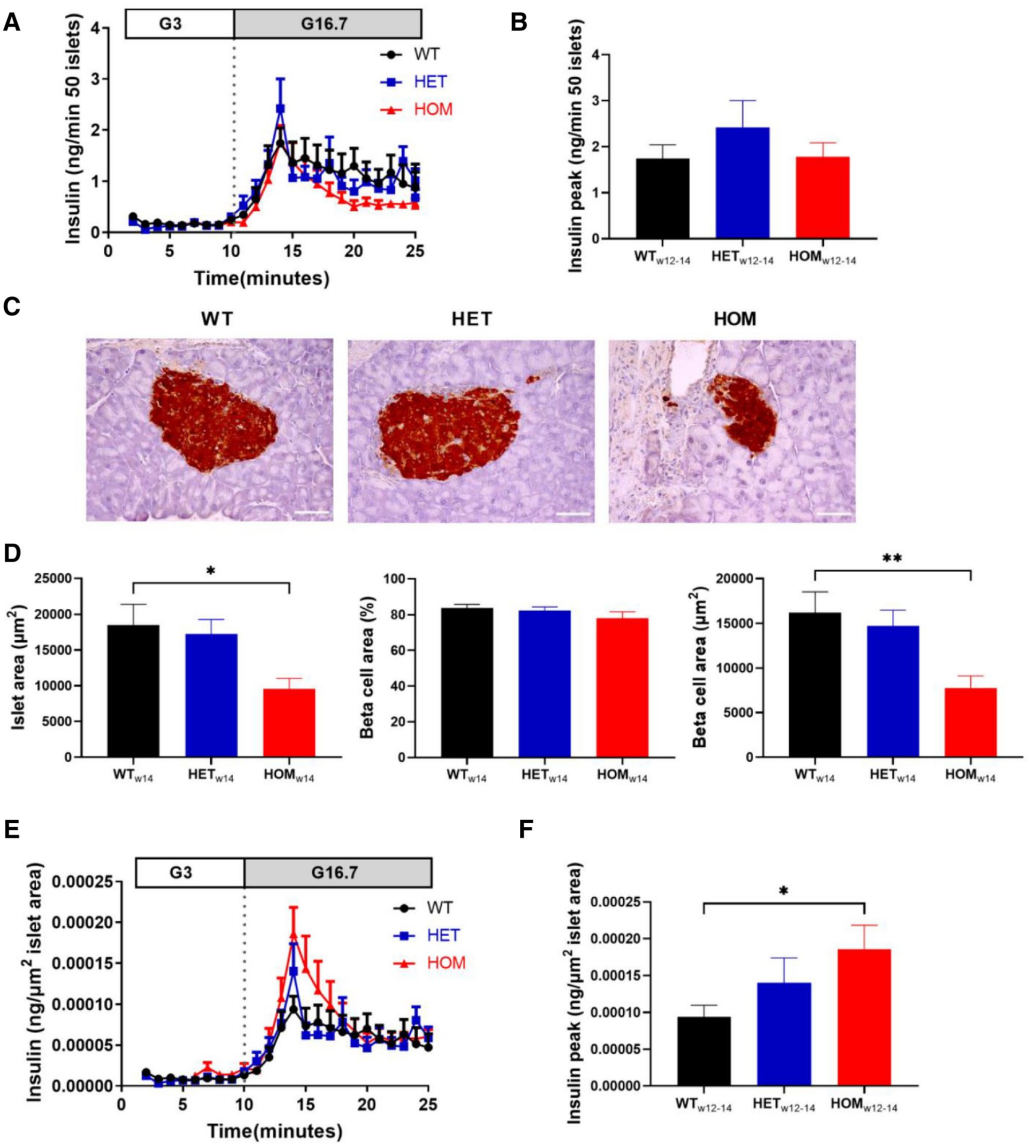
Ex vivo* g*lucose-stimulated insulin secretion and islet area in 12–14 week-old mice. (**A**) Isolated islets from 12 to 14 week old WT (n = 8), HET (n = 6) and HOM (n = 6) mice were stimulated with 3 and then 16.7 M glucose, and insulin was measured in the perifusate. (**B**) Peak insulin was compared. (**C**) Pancreatic sections of mice at 14 weeks of age were stained for insulin in WT (n = 6), HET (n = 7) and HOM (n = 7) mice, thereby staining beta-cells brown as shown in representative images (scale bar: 50 µm). (**D**) Islet area, percentage of beta-cell area and absolute beta-cell area were measured in 5 islets per mouse and subsequently averaged per mouse. (**E**) Insulin secretion curves from (**A**) normalized to islet area from (**D**) averaged per group, revealing increased peak insulin (**F**) in HOM. Tested with one-way ANOVA with Dunnett’s multiple comparisons testing. *: WT versus HOM, *P* < 0.05, **: WT versus HOM, *P* < 0.01. ANOVA, analysis of variance;HET, heterozygous; HOM, homozygous; WT, wild-type.

To investigate pancreatic islet insulin secretion in the absence of systemic influences, we isolated pancreatic islets from mice at 12–14 weeks of age and evaluated glucose-stimulated insulin secretion ex vivo. Total glucose-stimulated insulin secretion was comparable in WT, HET and HOM mice (Fig. 4A,B; peak insulin: *P* = 0.45). Interestingly, islets of HOM mice appeared noticeably smaller. To further investigate the islet and beta-cell area of these mice, we performed immuno-histochemical analysis of the pancreatic tissue of mice at 14–15 weeks of age (Fig. 4C). Although islet area of HET mice was comparable to WT (Fig. 4D; *P* = 0.68), islet area in HOM mice was significantly smaller than in WT littermates (*P* = 0.02 HOM vs WT). Beta-cell area as a percentage of whole islet area was not affected by genotype (*P* = 0.32), resulting in reduced absolute beta-cell area compared to WT in HOM mice (*P* = 0.0097), but not in HET mice (*P* = 0.59). Glucose-stimulated insulin secretion was therefore normalized to average islet area per genotype to account for islet size (Fig. 4E), revealing increased insulin secretion in these young HOM mice (Fig. 4F; *P* = 0.049).

### The increased ex vivo insulin secretion from islets from mice with *Kcnq1* A340V mutation devolved into hyposecretion with age

**Figure 5:**
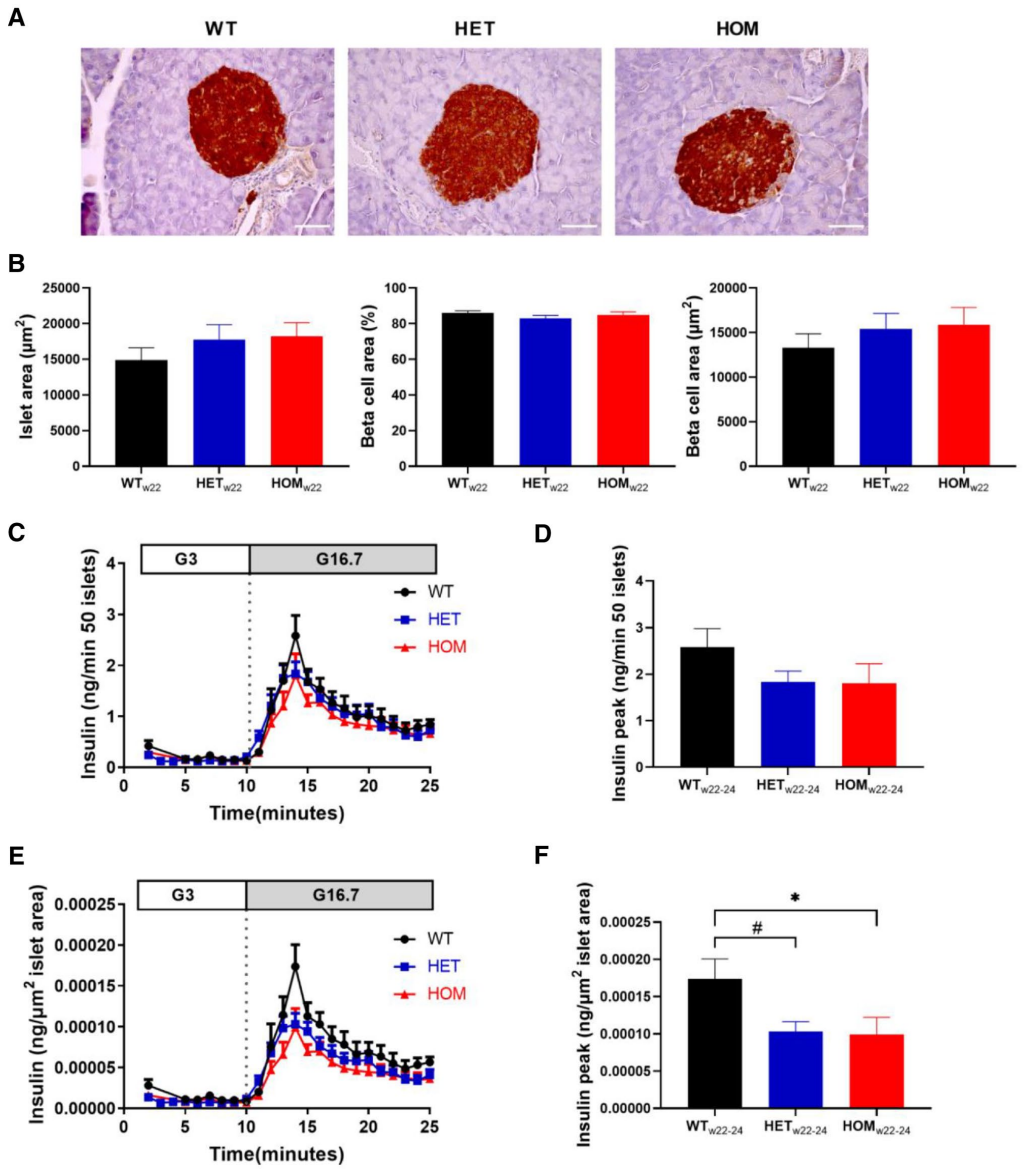
Ex vivo* g*lucose-stimulated insulin secretion and islet area in 22–24 week old mice. (**A**, **B**) Pancreatic sections of mice at 22 weeks of age were stained for insulin from WT (n = 6), HET (n = 9) and HOM (n = 7) mice, as shown in representative images (scale bar: 50 µm) (**A**). Islet area, the percentage of beta-cell area, and the absolute beta-cell area were measured in 5 islets per mouse and subsequently averaged per mouse. (**C**) Isolated islets from 22 to 24 week old WT (n = 7), HET (n = 9) and HOM (n = 8) mice were first exposed to 3 and then 16.7 M glucose. Insulin was measured in the perifusate and comparisons of peak insulin levels are depicted **(D)**. (**E**) Glucose-stimulated insulin secretion was normalized to islet area measured in (**B**) and comparisons of peak insulin levels are depicted (**F**). Tested with one-way ANOVA with Dunnett’s multiple comparisons testing. *: WT versus HOM, *P* < 0.05; #: WT versus HET, *P* < 0.05. ANOVA, analysis of variance; HET, heterozygous; HOM, homozygous; WT, wild-type.

**Figure 6:**
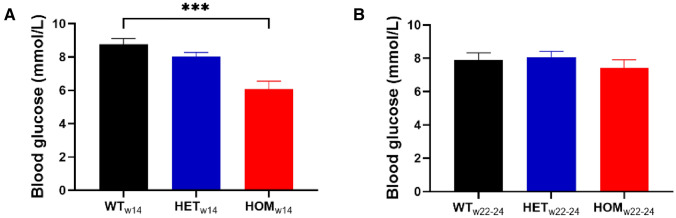
Non-fasting blood glucose. (**A**) Non-fasting blood glucose at 14 weeks of age in WT (n = 5), HET (n = 5) and HOM (n = 4) mice. (**B**) Non-fasting blood glucose at 22–24 weeks of age in WT (n = 6), HET (n = 14) and HOM (n = 8) mice. Tested with one-way ANOVA with Dunnett’s multiple comparisons testing. ***: WT versus HOM, *P* < 0.001.

Since we discovered that young HOM mice exhibited a hypersecretory phenotype, and since hypersecretion challenges the biosynthetic demand for insulin, we reasoned that with time these hyperactive beta-cells might experience difficulty meeting the increased biosynthetic and secretory demand. This concept is most clearly supported by the severe insulin-deficient phenotype of the Akita mouse that carries a mutation in the *ins-2* gene, causing misfolding of proinsulin, leading to ER stress, ER destruction and insulin deficiency^35^. Therefore we decided to repeat the histological examination and the islet perifusion after an 8–10 week follow-up period. At 22 weeks of age, islet area was comparable in all three genotypes (Fig. 5A,B; *P* = 0.5). Similarly, beta-cell area was also comparable in all three groups (Fig. 5B). Glucose-stimulated insulin secretion was assessed in islets at 22–24 weeks of age (Fig. 5C), showing comparable peak insulin secretion in islets from WT, HET and HOM mice (Fig. 5D; *P* = 0.3). However, correction to islet area (Fig. 5E) revealed decreased peak insulin secretion in HET (*P* = 0.049) and HOM (*P* = 0.04) islets compared to WT (Fig. 5F). Thus, normalized glucose-induced insulin secretion increased in WT, decreased in HET, and further decreased in HOM islets over time (compare Figs. 4E with 5E). To check whether an age difference was evident in vivo, non-fasting blood glucose was measured prior to islet or pancreas isolation in a subset of mice. At 14 weeks of age, non-fasting blood glucose was comparable in WT and HET mice (Fig. 6A; *P* = 0.262), but reduced in HOM mice (*P* = 0.0006 compared to WT). At 22–24 weeks of age, on the other hand, non-fasting blood glucose was comparable in all groups (Fig. 6B; *P* = 0.5), further supporting an age-dependent change.

## Discussion

In this study, we present a novel mouse model of LQTS carrying the LoF mutation *Kcnq1*-A340V, as identified in patients. Mice homozygous for this mutation had increased QT interval, which further prolonged after refeeding. Although in vivo insulin response to oral glucose was not different to that of littermate controls, islets from young mice carrying this mutation secreted more insulin when exposed to glucose ex vivo*.* Additionally, their non-fasting blood glucose was reduced. This is in line with previous in vivo data from LQTS patients with LoF *KCNQ1* mutations^12^. Interestingly, at this time-point, HOM mice had smaller islet and beta-cell areas compared to WT. This could suggest that the hypersecretory beta-cell phenotype requires less beta-cell mass to yield equivalent insulin secretion in young mice, or could reflect reduced beta-cell proliferation during development, potentially through epigenetic regulation of *Cdkn1c,* as shown for a mutation in the non-coding region of *Kcnq1*^24^. The hypersecretory phenotype disappeared with age, however, and devolved into hyposecretion compared to islets from littermate controls. At follow-up 8–10 weeks later, the islet and beta-cell areas were comparable in all three groups of mice. At this time, glucose-stimulated insulin secretion was in fact reduced in islets from both HET and HOM mice, revealing a transition from hyper- to hyposecretion of insulin in the setting of K_v_7.1 dysfunction.

Recently, Yang et al*.* reported a transition from hyper- to hypoinsulinemia secondary to dysfunction of K_v_11.2, another voltage-gated K^+^ channel, encoded by *Kcnh6*^36^. Islets from 3-week-old *Kcnh6* KO mice exhibited increased insulin secretion and associated increased intracellular Ca^2+^ concentrations. However, islets from 30-week-old *Kcnh6* KO mice exhibited increased intracellular Ca^2+^, along with increased levels of endoplasmic reticulum (ER) stress and apoptosis, resulting in beta-cell loss and decreased insulin secretion^36^. As prolonged increased intracellular Ca^2+^ can be toxic to the cell^37^, and increased insulin demand can result in ER stress, the authors suggested that hypersecretion can eventually lead to beta-cell failure, apoptosis and beta-cell loss^36^. In the present study, hypersecretion of insulin coincided with lower non-fasting blood glucose levels in HOM mice at 14 weeks of age despite lower beta-cell mass. Chronic high insulin biosynthetic demand could lead to ER stress and an eventual reduction in insulin secretion, as seen during follow-up at 22–24 weeks of age. This decrease in insulin secretion, in combination with the hyperactivity-related high sympathetic activity, may drive beta-cell growth to compensate for the ensuing higher blood glucose excursions, which could explain comparable beta-cell area in the HOM mice at that time. The transition from increased glucose-stimulated insulin secretion to subsequent hyposecretion possibly results from hypersecretion-induced islet dysfunction. This may explain the association to both post-prandial hypoglycemia in LQTS patients with LoF variants^11^, as well as increased risk of T2D-associated with *KCNQ1* variants^6,21,22^ and the higher prevalence of diabetes in LQTS patients^23^.

In our hands, HOM mice did not have a decreased HOMA-IR index. Previously, *Kcnq1* KO mice have been reported to have lower fasting blood glucose and plasma insulin^27^. Peripheral glucose uptake in skeletal muscle was increased in the *Kcnq1* KO mice compared with WT mice, suggesting that increased insulin sensitivity was responsible for the lower insulin levels^27^. However, we show increased physical activity in HOM mice, which could also be responsible for the increased uptake of glucose in skeletal muscle. The hyperactive phenotype of the HOM mice has previously been described in *Kcnq1* KO mice and is referred to as a ‘shaker/waltzer’ phenotype thought to be the result of functional loss of K_v_7.1 in the inner ear^38^. The phenotype is characterized by hyperactivity, rapid head movements and periodic, rapid circling, traits all observed in our HOM mice. This phenotype was aggravated by exogenous stressors, such as sampling and handling. Although human LQT1 patients with an autosomal recessive form (Jervell and Lange-Nielsen syndrome (JLNS)) have congenital hearing loss, they do not exhibit hyperactive behaviour^39^. The hyperactive behaviour of the mice, along with possible higher insulin sensitivity, complicates the interpretation of oral glucose-induced insulin secretion in vivo*.* To what extent JLNS patients exhibit post-prandial hyperinsulinemia and subsequent hypoglycaemia is unknown.

Acute hyperglycemia increases QT-interval^40^, which could be more detrimental in patients with pre-existing long QT. In fact, an acute increase in blood glucose prolongs QT-interval and alters T-wave morphology in healthy controls and in LQT1 patients and are more pronounced in the latter^14^. In the present study, we show that refeeding increases QT interval in HET and HOM mice. Hyperglycemia has been shown to reduce K_v_11.1 current through production of reactive oxygen species^41^. In the setting of diminished repolarization reserve due to reduced K_v_7.1 current, any reduction of K_v_11.1 will be more pronounced. The further prolongation of QT during hyperglycemia could pose a further risk for LQT1 patients and could potentially lead to arrhythmia^42^. Here we show that in HOM mice, refeeding not only increased QT, but also increased the number of PVCs, suggesting that metabolic challenges may evoke ventricular repolarization disturbances in LQT1 that increase the risk of arrhythmias.

The present study has some limitations. The in vivo data are confounded by the high activity level exhibited by the HOM mice. Sympathetic nervous system  activity and catecholamine release, which are expected to be high at these activity levels, inhibit insulin secretion^43^. Blocking this sympathetic activity during an OGTT, by pharmacological blockade of the α- and β-adrenergic receptors, may reveal the beta-cell phenotype in vivo*.* Glucose uptake in the gut during the OGTT could be affected as K_v_7.1 is expressed in the stomach, intestine and exocrine pancreas^44,45^. Intra-peritoneal glucose tolerance tests could be conducted to bypass gastrointestinal confounding in future studies. LQT1 patients heterozygous for A341V present with a clinical severity compatible with a dominant-negative mode of action, which was not recapitulated in our HET mice, possibly due to differences in epigenetic modifications or species differences in the turn-over of the mutant protein. Follow-up experiments were only performed 8–10 weeks after initial experiments. It would be of interest to follow islet development, as well as glucose-stimulated insulin secretion, over a longer span of time and in response to other metabolic challenges, such as a high fat diet. Finally, caution is advised when translating mouse data to the clinic, especially with regard to the cardiac phenotype, given the low repolarization dependence on *KCNQ1* in adult murine hearts compared to humans, and in light of the in vivo hyperactivity phenotype exhibited by these mice that is not reported in the clinic.

In conclusion, ex vivo glucose-stimulated insulin release was increased in islets from HOM mice at 12–14 weeks of age, confirming beta-cell-intrinsic regulation of insulin secretion by K_v_7.1. Interestingly, during follow-up at 22–24 weeks of age, ex vivo insulin secretion was reduced in islets from HET and HOM mice compared to WT. Between these two age points, insulin secretion had increased in WT, decreased in HET mice and further decreased in HOM mice, indicative of progressive exhaustion. These data suggest that dysfunction of K_v_7.1 is involved in a transition from hyper- to hyposecretion of insulin, possibly explaining the association with both hypoglycemia and hyperglycemia in LQT1 patients.

## Methods

### Mouse model

The full-length mouse and human amino acid sequences for *Kcnq1* isoform 1 share > 88% overall amino acid identity and > 91% amino acid conservation^46^. The A341 residue in humans corresponds to the A340 residue in the mouse, which in our study was mutated to V to mimic the human mutation. This human mutation is common amongst LQT1 patients and causes a severe phenotype^30^. Single guide (sg) RNAs were designed by the online tool: https://zlab.bio/guide-design-resources (Table S1) and were cloned into the backbone vector pSpCas9(BB)-2A-Puro (PX459) V2.0 (a gift from Feng Zhang; Addgene plasmid # 62988^28^) by using golden-gate sgRNA cloning protocol^47^. Correct insertions were confirmed by sequencing. The targeted cleavage efficiency was tested by the T7 endonuclease I^48^, and indel mutation efficiency = 100 × (1 –  (1 –  (b + c)/(a + b + c))^1/2^)^28^ was used for the sgRNA selection. Six sgRNAs (Supplemental Table S1) were selected and their efficiency was tested in the mouse cell line NIH-3T3 (Supplemental Figure S1). Due to its high efficiency and position next to the target mutation, sgRNA5 was chosen for further experimentation. The asymmetric repair template was designed according to Corn et al.^49^: 5’-TCTCTGTCTTCGCCATATCCTTCTTTGTTCTACCC**GCAGTG**GGTACCTATTAAGAGC CCTTTCCCTTGTTTCCCACTCACCCTTGCCCCAACTTCTAGGCATTCCTTGAAGTAGAGTAAGGAGGCCT-3’, where a missense mutation (A340V, underlined) and a silent mutation (introducing a restriction site for Bts^α^I, in bold) have been inserted as illustrated in Figure S2A. The mutated embryo stem cells (ESCs) were generated by electroporating hybrid 129, C57Bl/6 ES cells with the Cas9/sgRNA5 vector and its corresponding repair template. After puromycin selection, single ESC clones were screened by Bts^α^I digestion (Figure S2B), and the correct mutation insertion was confirmed by sequencing. To generate mutant mouse chimeras, a homozygous ESC clone with correct insertion was injected into C57BL/6 blastocysts. The sequences of the chimeras were confirmed by sequencing (Figure S2C). The HET male chimeras were crossed with C57BL/6 WT females, and genotypes were confirmed by Bts^α^I digestion. Males and females positive for the mutation from the F1 pups were inter-crossed, generating WT, HET and HOM animals for the experiments. RT-qPCR showed expected distribution of WT and mutant mRNA in the left ventricle of the heart (Figure S4).

Mice were housed in a temperature-controlled environment (22 ± 1 °C) with 12-h light/dark cycles and had access to chow (1310, Altromin, Lage, Germany) and water ad libitum. All procedures were carried out in accordance with Danish guidelines and regulations. The breeding of the mouse strain was approved by the *Danish Working Environment Authority* with permit 20180030710/4. The experiments were approved by *The Danish Animal Experiments Inspectorate* permit numbers 2015-15-0201-00728, 2018-15-0201-01397, 2018-15-0201-01558 and 2018-15-0201-01584 and reported in accordance with the ARRIVE guidelines.

### Genotyping

DNA samples were extracted from ESCs by salt precipitation method^50^ or lysed from mouse tissues by alkaline extraction^51^. The concentration of DNA in samples was quantified by NanoDrop spectrophotometry (Thermo Scientific, Massachusetts, United States), and DNA was used for PCR amplification using specific primers (Table S2) and HotStarTaq DNA polymerase, following the manufacturer's instructions. Genotyping was performed by digestion of the PCR amplification product using the restriction enzyme Bts^α^I.

### Quantitative real-time PCR

RNA was extracted using the NucleoSpin RNA kit (Macherey-Nagel, PA, USA) and RNA quality and concentration checked on NanoDrop2000 (ThermoScientific, Denmark). cDNA was synthesized using the iScript cDNA Synthesis kit (Bio-Rad, Denmark) and used for RT-qPCR using SYBR Green Master Mix (Applied Biosystems, Denmark). Data was acquired using the QuantStudio 5 Real-Time PCR system (Applied Biosystems, Denmark). Primers were purchased from TAG Copenhagen (Denmark). For primer sequences, see Table S3.

### Electrocardiography

Male mice at 9–12 weeks of age were anesthetized (2% isoflurane in 100% O_2_) and placed in a supine position on a heating pad. Body temperature was closely monitored and kept at 37.0 ± 0.5 °C (two mice were excluded due to failure to maintain body temperature). Subcutaneous needle electrodes were used to record a 6-lead surface electrocardiogram (ECG)^52^ at 4 kHz using LabChart 8 (ADInstruments, Australia). After 5 min of baseline recording, mice received an intraperitoneal injection of isoprenaline (200 µg/kg), and the ECG was recorded for 15 min. Isoprenaline was administered to further exacerbate the long QT phenotype, as described by Knollmann et al.^46^, as *I*_Ks_ is stimulated by beta-adrenergic activation^44^. QT correction for heart rate is not recommended in mice, especially not under anaesthesia^53^, and was therefore not performed.

### Oral glucose tolerance test

Prior to the OGTT, male 9–11-week-old mice were fasted five hours before being given oral gavage with 2 g/kg glucose. Blood glucose was measured after tail tip punctures using glucometer (Accu-check compact plus, Roche, Switzerland), at times 0, 15, 30, 45, 60, 75 and 90 min after the glucose load. Simultaneously, blood samples were taken from the retrobulbar plexus at times 0, 15, 30, 60 and 90 min, and plasma samples were transferred to new tubes after 20 min and centrifuged at 2800 g at 4 °C. Insulin levels were subsequently measured by mouse insulin ELISA (Mercodia AB, Sweden) according to the manufacturer's instructions, with a detection limit of 2 ng/ml. The plasma samples from the OGTT and the samples from islet perifusion (see below) were measured without dilution.

### Glucose telemetry

In a subset of mice, blood glucose was measured continuously using the Physiotel HD telemetry system with the HD-XG implants (Data Sciences International, USA). For the device implantation surgery, the mice were anesthetized with isoflurane in 100% O_2_ (2.5% for induction, 1.5% for maintenance). Lidocaine (7 mg/kg) and Carprofen (10 mg/kg) were administered preoperatively for local and general analgesia, respectively. All surgical procedures were conducted using strictly aseptic techniques. The catheter on the HD-XG was inserted in the left common carotid artery, and advanced to the point in the aortic arch where the tip containing the enzymatic sensor was placed. The transmitter body was placed in a subcutaneous pocket on the right flank, and the incision was closed with resorbable suture (7–0 Vicryl, Ethicon, USA). After surgery the mice were placed in clean cages for recovery, and thermal support was provided for the first 24 h post-operation by placing the cages partially on a 37 °C heat pad. Device placement surgeries were done in 13–15-week-old male mice. After one week of recovery from surgery, telemetry devices were calibrated according to the manufacturer’s instructions. Subsequently, an OGTT was performed, dosing 2 g/kg glucose. Data were collected using Ponemah Version 6.41 (Data Sciences International, USA).

### Electrocardiogram telemetry

Continuous ECG monitoring was carried out via implanted telemetry devices in awake, freely moving mice. Mice (female, 14–15 weeks of age) were anesthetized using 2% isoflurane in 100% O_2_ and body temperature was maintained at 37 ± 0.5 °C. For post-operative analgesia, carprofen (10 mg/kg) was pre-operatively injected subcutaneously. Subsequently, a subcutaneous pocket was made in the mid-dorsal region, a mixture of lidocaine (0.4 mg/kg) and bupivacaine (1 mg/kg) was administered in the pocket, and the telemetry device was placed inside. Leads were tunneled subcutaneously and sutured on the muscle in a lead II configuration using non-absorbable suture. After surgery, mice were placed in clean cages for recovery. After 2 weeks of recovery, continuous ECG recordings were made during an 18 h fast, with subsequent refeeding by reintroduction of standard chow food into the cage.

### Islet isolation and incubation

Pancreatic islets were isolated from male mice after injection of liberase (0.1 mg/mL dissolved in Hank's Balanced Salt Solution) into the common bile duct. The pancreas was subsequently immersed in a water bath at 37 °C, shaken and then placed on ice. Three washing rounds were conducted by collecting the supernatant after 5 min of incubation on ice and replacing the salt solution. Islets were then handpicked under a stereo microscope (Leica). The isolated islets were incubated at 37 °C in a 5% CO_2_ incubator, with 11 mM glucose RPMI medium supplemented with 10% fetal bovine serum and 1% penicillin/streptomycin. The medium was changed the day after isolation.

### Glucose-stimulated insulin secretion ex vivo

The islet perifusion system has been described previously^54,55^. After 24–48 h of incubation, fifty islets per well were pre-incubated in 2.8 mM glucose RPMI medium for 2 h prior to perifusion. Islets were subsequently perifused with Krebs Ringer Salt Solution buffer (135 mM NaCl, 3.6 mM KCl, 0.5 mM NaH_2_PO_4_, 0.5 mM MgCl_2_, 2.5 mM CaCl_2_, 2 mM NaHCO_3_, 10 mM HEPES, 1 g/L BSA, pH = 7.4) containing 3 mM glucose at a flow rate of 1 mL/min. After 15 min of stabilization, samples taken every min for 9 min were used for baseline. The islets were then perifused with 16.7 mM glucose buffer for 15 min, and samples were taken every min. After the experiment, islets were collected, counted and washed twice with cold PBS buffer and then lysed in 1 mL 70% ethanol–HCl at -20 °C for at least 24 h. Insulin in the perifusates was measured as mentioned above.

### Immunohistochemistry for visualization of insulin

The dissected pancreata were fixed in 4% paraformaldehyde, embedded in paraffin and cut in 5 µm sections for insulin staining. The samples were incubated overnight at 40 °C with the primary in-house antibody Insulin 2006-4 (provided by JJ Holst), followed by a 40 min incubation with biotinylated goat anti-guinea pig IgG antibody (Vector Laboratories, Peterborough, United Kingdom). Subsequently, a 30 min avidin/biotin ABC complex formation was obtained using Vectastain ABC HRP kit (Vector Laboratories, Peterborough, United Kingdom) followed by a 15 min incubation with 3,3–diaminobenzidine (Vector Laboratories, Peterborough, United Kingdom).

Blinded to genotype, five representative islets per mouse were imaged using a 20 × or 40 × objective (one mouse was excluded due to lack of islets in section). Islet area and beta-cell area were subsequently estimated by Image Pro Plus 7.0, also blinded to genotype, by a second observer. Fractional beta-cell area was calculated as the total area of insulin-positive beta-cells divided by the total islet area. Measurements were averaged per mouse.

### Data analysis

ECG analysis was performed offline, blinded to genotype. One minute periods of ECG recordings were used to create a signal-averaged ECG aligned at the R peak. ECG parameters were subsequently measured manually, as previously described in detail^53^. The end of the QRS complex was determined by the change in slope after the S deflection and the T wave was determined using the tangent method^53^. For the 6-lead ECG recorded under anaesthesia, each ECG parameter was measured separately in lead I-III, and the longest measure was used for comparative analysis. For OGTT incremental areas under the curve (AUC) were calculated using Prism Version 7.05 (GraphPad Software, California, United States). The homeostasis model assessment-estimated insulin resistance (HOMA-IR) was calculated using the formula HOMA-IR = fasting insulin (uIU/mL) × fasting glucose (mmol/L)/22.5. Data from glucose telemetry were analysed using Microsoft Excel 2016.

### Statistics

Statistical analyses were performed using Prism (Prism 7, GraphPad Software Inc., La Jolla, USA). Data were tested for normality (Shapiro-Wilk). To study the interaction between one independent variable on the dependent variable between multiple groups, data were tested using a one-way analysis of variance (ANOVA) with multiple comparisons testing (Dunnett’s) to WT for parametric testing, or by Kruskal–Wallis test with Dunn’s multiple comparison testing to WT for nonparametric testing. QT/RR relationships were compared with linear regression analysis. Data are shown as mean ± standard error of the mean (SEM). *P* values < 0.05 were considered significant.

## Supplementary Information

Supplementary Information.

## Abbreviations

ANOVA: Analysis of variance
AUC: Area under the curve
Cdkn1c: Cyclin-dependent kinase inhibitor 1C
CRISPR: Clustered regularly interspaced short palindromic repeats
ECG: Electrocardiogram
ER: Endoplasmic reticulum
GWAS: Genome-wide association study
HET: Heterozygous mouse
HOM: Homozygous mouse
HOMA-IR: Homeostasis model assessment-estimated insulin resistance
*I*_Ks_: Slow delayed rectifier potassium current
KO: Knock-out
K_v_: Voltage-gated potassium channel
LoF: Loss-of-function
LQT1: Long QT syndrome 1
LQTS: Long QT syndrome
OGTT: Oral glucose tolerance test
PVCs: Premature ventricular contractions
sgRNA: Single guide ribonucleic acid
T2D: Type 2 diabetes
WT: Wild type
