# Supplementary material for: Age-dependent transition from islet insulin hypersecretion to hyposecretion in mice with the long QT-syndrome loss-of-function mutation Kcnq1-A340V
Source: Sci Rep. 2021 Jun 10;11:12253. doi: 10.1038/s41598-021-90452-8 (PMC8192901; doi:10.1038/s41598-021-90452-8)
Supplement: Supplementary file 1 — Supplementary Information. [file 41598_2021_90452_MOESM1_ESM.pdf]

SUPPLEMENTAL MATERIAL

Age-dependent transition from islet insulin hypersecretion to hyposecretion in mice with the long QT-syndrome loss-of-function mutation Kcnq1-A340V

Anniek F. Lubberding[1], Jinyi Zhang[1,2], Morten Lundh[2], Thomas Svava Nielsen[2], Mathilde S. Søndergaard[1], Maria Villadsen[1], Emil Z. Skovhoj[1], Geke A. Boer[1,2], Jakob Bondo Hansen[2], Morten B. Thomsen[1], Jonas T. Treebak[2], Jens J. Holst[1,2] , Jørgen K. Kanters[1], Thomas Mandrup-Poulsen[1], Thomas Jespersen[1], Brice Emanuelli[2], and Signe S. Torekov[1,2]

1. Department of Biomedical Sciences, Faculty of Health and Medical Sciences, University of Copenhagen, Copenhagen, Denmark;

2. Novo Nordisk Foundation Center for Basic Metabolic Research, Faculty of Health and Medical Sciences, University of Copenhagen, Copenhagen, Denmark

Corresponding author:

Signe Sørensen Torekov, Department of Biomedical Sciences, Faculty of Health and Medical Sciences, University of Copenhagen, Blegdamsvej 3B, DK-2200, Copenhagen, Denmark,

Email: torekov@sund.ku.dk

Supplemental methods

Reagents

REAGENT	SOURCE
	Cloning and Transfection
**The backbone vector pSpCas9(BB)-2A-Puro (PX459) V2.0**	Addgene (Cambridge, MA, USA)
**Oligo DNAs listed in Supplementary TableS1 (4-μmol ultramers)**	TagCopenhagen (Copenhagen, Denmark)
**One Shot Stbl3 chemically competent *E. coli***	Life Technologies
**Rapid ligase buffer, 2×**	Enzymatics (Copenhagen, Denmark)
**T7 ligase**	Enzymatics (Copenhagen, Denmark)
**T4 polynucleotide kinase**	New England BioLabs (Herlev, Denmark)
**T4 DNA ligase reaction buffer, 10×**	New England BioLabs (Herlev, Denmark)
**BSA enzyme**	New England BioLabs (Herlev, Denmark)
**Bbsl**	New England BioLabs (Herlev, Denmark)
**HiSpeed Plasmid Maxi Kit**	Qiagen (Copenhagen, Denmark)
**NIH-3T3 cells**	ATCC (Wesel, Germany)
**TransIT transfection reagent**	Mirus (Madison, US)
**The repair template (oligo DNA)**	Integrated DNA technologies (Leuven, Belgium)
	Sequencing
**QIAquick gel extraction kit**	Qiagen (Copenhagen, Denmark)
**QIAprep spin miniprep kit**	Qiagen (Copenhagen, Denmark)
**TOPO TA Cloning Kit**	Thermo Fisher Scientific (Slangerup, Denmark)
	DNA extraction and analysis

**Proteinase K**	Sigma-Aldrich
**Accutase**	Sigma-Aldrich
**Tris-EDTA(TE) buffer**	Amresco
**Oligo DNAs**	TagCopenhagen (Copenhagen, Denmark);
**HotStarTaq DNA Polymerase**	Qiagen (Copenhagen, Denmark);
**dNTP mix**	Thermo Fisher Scientific (Slangerup, Denmark);
**T7 endonuclease I**	New England BioLabs (Herlev, Denmark);
**Bts[a]I**	New England BioLabs (Herlev, Denmark);
**CutSmart Buffer 10×**	New England BioLabs (Herlev, Denmark);
Islet isolation	
**Hanks' Balanced Salt solution + Ca^{2+} and Mg^{2+} with no phenol red**	Sigma-Aldrich (Denmark)
**Liberase TL**	Roche (Basel, Switzerland)
Chemicals	
**Isopropanol**	Sigma-Aldrich (Denmark)
**NaCl**	Sigma-Aldrich (Denmark)
**99% Ethanol**	Sigma-Aldrich (Denmark)
**KCl**	Sigma-Aldrich (Denmark)
**NaH_2PO_4**	Sigma-Aldrich (Denmark)
**$MgCl_2$,**	Sigma-Aldrich (Denmark)
**$CaCl_2$**	Sigma-Aldrich (Denmark)
**$NaHCO_3$**	Sigma-Aldrich (Denmark)
**HEPES**	Sigma-Aldrich (Denmark)
**BSA**	Sigma-Aldrich (Denmark)

	Cell and islet culture media
**D-Glucose solution**	Thermo Fisher Scientific (Slangerup, Denmark);
**DMEM-high glucose,**	Thermo Fisher Scientific (Slangerup, Denmark);
**RPMI medium 1640 + Glutamax**	Thermo Fisher Scientific (Slangerup, Denmark);
**RPMI medium 1640 - D-glucose**	Thermo Fisher Scientific (Slangerup, Denmark);
**Penicillin-streptomycin**	Thermo Fisher Scientific (Slangerup, Denmark);
**Fetal bovine serum (FBS)**	Sigma-Aldrich (Denmark)
	Insulin measurement
**Mouse insulin ELISA kit**	Mercodia (Uppsala,Sweden)

Supplemental tables:

Table S1 sgRNAs designed by web tool.

No.	Forward	Reverse
sgRNA1	5'-CACCGTCTTAATAGGTACCCACCGC-3'	3'-CGCGGTGGGTACCTATTAAGACAAA-5'
sgRNA2	5'-CACCGCTTAATAGGTACCCACCGCT-3'	3'-CAGCGGTGGGTACCTATTAAGCAAA-5'
sgRNA3	5'-CACCGCACCGCTGGGAGTGCAAAGA-3'	3'-CTCTTTGCACTCCCAGCGGTGCAAA-5'
sgRNA4	5'-CACCGATCCTTCTTTGCACTCCCAG-3'	3'-CCTGGGAGTGCAAAGAAGGATCAAA-5'
sgRNA5	5'-CACCGCTTCTTTGCACTCCCAGCGG-3'	3'-CCCGCTGGGAGTGCAAAGAAGCAAA-5'
sgRNA6	5'-CACCGTTCTTTGCACTCCCAGCGGT-3'	3'-CACCGCTGGGAGTGCAAAGAACAAA-5'

Table S2 Primer list for genotyping

No.	Forward primer	Reverse primer
1	5'- ACTACCATTGGCTACGGGGA -3'	5'-TGCCGCTTCTGTGAAGTACC -3'
2	5'-CTTCCTTGCAGGTCACAGTCA-3'	5'-CCACAACAAGGCCTCCTTAC-3'

Table S3 Primer list for RT-qPCR

Gene	Forward primer	Reverse primer
KCNQ1_WT	5'- GCCATATCCTTCTTTGCACTC-3'	5'- AAGTGCTTCTGCCTCTGCTT-3'
KCNQ1_mutant	5'- TCGCCATATCCTTCTTTGTTC-3'	5'- AAGTGCTTCTGCCTCTGCTT-3'
KCNQ1_both	5'- TTGGAAGTGTTTCGTGTACCA-3'	5'- ACTGAAGATGAGGCAGACCAG-3'
RPL32	5'- AGAAGTTCATCAGGCACCAGT-3'	5'- TTTCTTGTTGCTCCCATAACC-3'

Supplemental Figures:

Figure S1 Efficiency test of sgRNAs

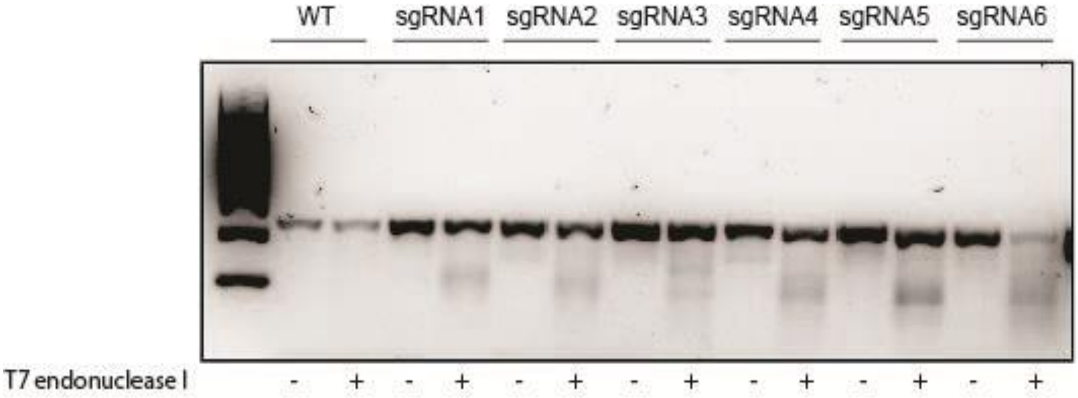

No.	sgRNA1	sgRNA2	sgRNA3	sgRNA4	sgRNA5	sgRNA6
Indel mutation %	2,15	1,94	4,75	6,98	13,94	10,10

Figure S2

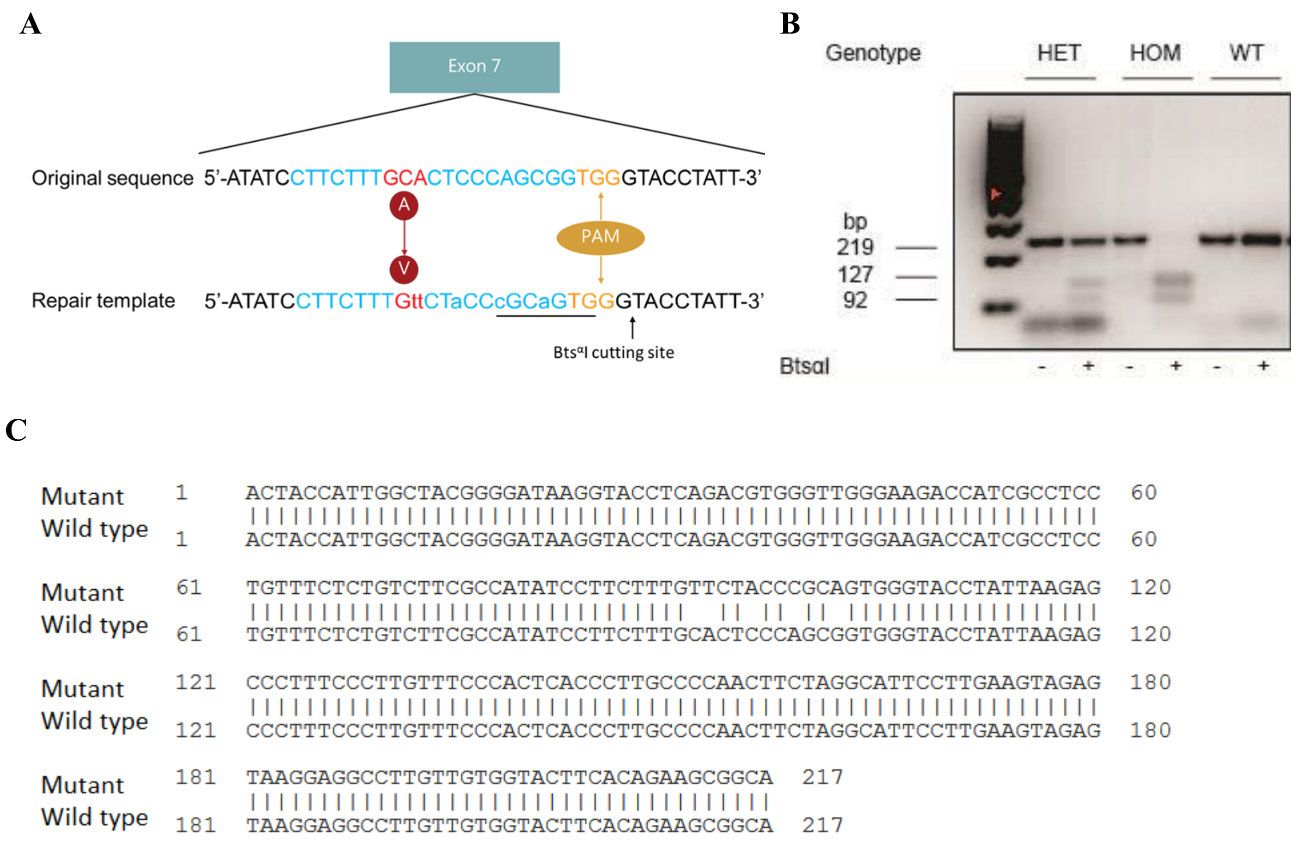

Figure S2. Generation of *KCNQ1*^{A340V} mice. A) In order to introduce the missense mutation *KCNQ1*^{A340V}, GCA was changed to GTT in the repair template, and a BtsαI cutting site (5'-GCAGTG-3') was introduced for genotyping. The altered nucleotides in the repair template are indicated as lowercase letters, and the protospacer adjacent motif (PAM) sequence is marked in yellow. **B)** Genotyping by PCR amplification of the region of interest (219bp) followed by digestion with restriction enzyme Bts^{a}I, resulting in two bands (127bp + 92bp) upon correct insertion of the repair template. PCR products would be fully digested from homozygous samples, half-digested from heterozygotes, and not digested from WT samples. **C**) Sequencing results confirmed the correct insertion of the mutations.

Figure S3

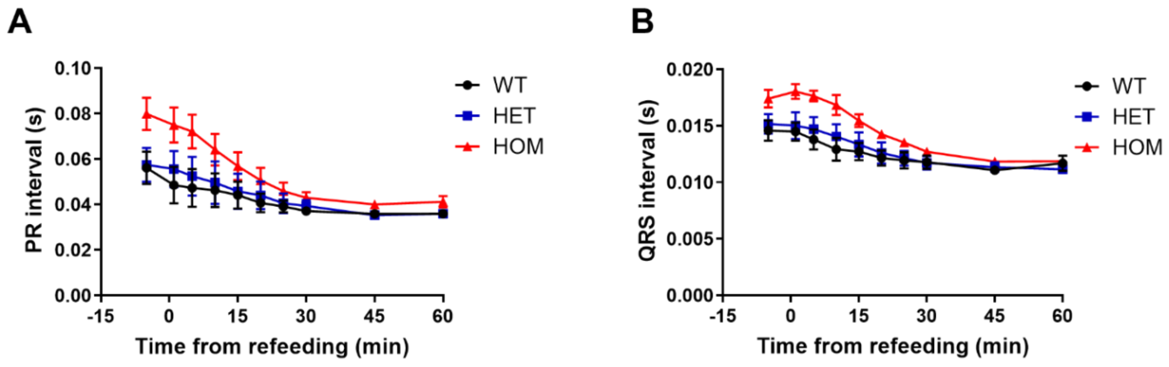

Figure S3. PR and QRS interval during refeeding. WT (n=8), HET (n=7) and HOM (n=4) mice were fasted overnight for 18 hrs, after which they were re-fed. **A&B**) PR interval (**A**) and QRS interval (**B**) dropped immediately upon refeeding.

Figure S4

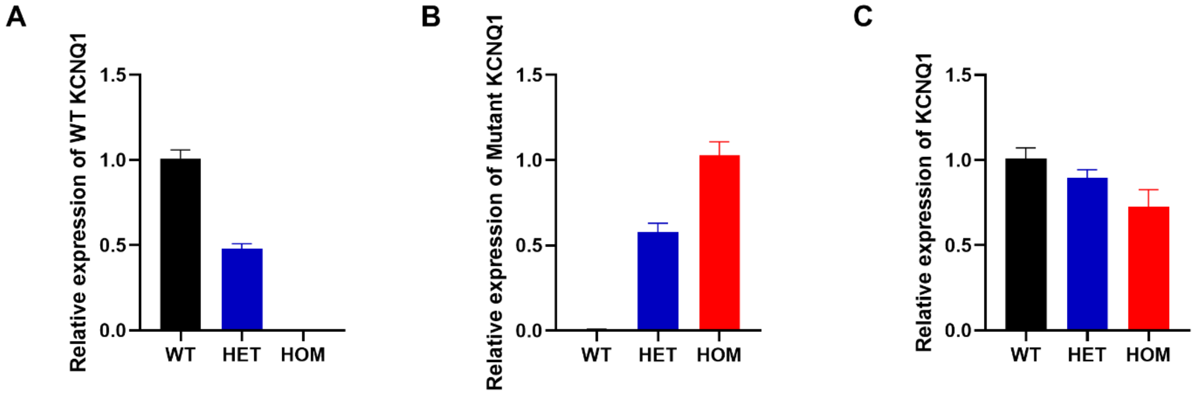

Figure S4. Relative expression of KCNQ1 mRNA in cardiac tissue. A-C) Relative expression ($2^{-\Delta\Delta CT}$) measured using Rpl32 as housekeeping gene in left ventricular tissue of WT (n=7), HET (n=14) and HOM (n=10) mice: WT KCNQ1 relative to average Ct values of WT (**A**), mutant KCNQ1 relative to average Ct values of HOM (**B**) and overall KCNQ1 mRNA (both WT and mutant) relative to average Ct values of WT (**C**).
